# Using Population-Level Data to Assess Need for and Use of Long-Term Services and Supports in California

**DOI:** 10.1093/geroni/igab046.1936

**Published:** 2021-12-17

**Authors:** Kathryn Kietzman, Lei Chen, Rebecca Allen

**Affiliations:** 1 University of California, Los Angeles, Center for Health Policy Research, UCLA Center for Health Policy Research, California, United States; 2 University of California, Los Angeles, Los Angeles, California, United States; 3 California State University, Long Beach, Long Beach, California, United States

## Abstract

In response to aging and disability stakeholder advocacy in California, the state’s 2018-19 budget included support for the development of a study of Californians with needs for long-term services and supports (LTSS). Existing data on LTSS most typically represents those who already use specific programs or services. Yet many programs do not uniformly collect and report data, or have little capacity to share data across different delivery systems. In response to these gaps, we developed a 15-minute follow-on survey to the 2019-2020 California Health Interview Survey (CHIS), gathering statewide population-level data to assess LTSS needs and use by Californians 18 years of age and older. This paper reports on preliminary findings from the 2019 CHIS-LTSS survey conducted with a sample of 1097 respondents. Screening questions identified respondents reporting difficulties with concentrating, remembering, or making decisions (60%), performing basic daily activities such as dressing or bathing (26%), or getting out of the house to shop or to see the doctor (52%). Nearly half of respondents (45%) reported needing help with routine care needs while 16% needed help with personal care needs. Additional findings illustrate specific LTSS needs, service use, consequences of unmet needs, financial concerns, and consumer experiences. At a time when California policy makers, program planners, and advocates are engaged in implementing a 10-year Master Plan for Aging, these findings can be used to identify and address gaps in the types of services and supports that are essential to meet the LTSS needs of older adults and people with disabilities.

